# Demographic, Health, and Exposure Risks Associated With Cognitive Loss, Alzheimer's Disease and Other Dementias in US Military Veterans

**DOI:** 10.3389/fpsyt.2021.610334

**Published:** 2021-02-25

**Authors:** Carolyn W. Zhu, Mary Sano

**Affiliations:** ^1^Icahn School of Medicine at Mount Sinai, New York, NY, United States; ^2^James J. Peters VA Medical Center, Bronx, NY, United States

**Keywords:** Alzheimer's disease, dementia, veterans, risk, risk management

## Abstract

The US military veteran population receiving care through the Veterans Health Administration (VHA) is particularly susceptible to cognitive impairment and dementias such as Alzheimer's disease and related dementias due to demographic, clinical, and economic factors. In this report we summarize the prevalence of dementia among US veterans and risks associated with AD and related dementias. We discuss the likelihood that these risks may be increasing in those about to enter the age in which dementias are common. We propose that VHA, the largest integrated health care system in the US, has shown promise in managing health risks that impact dementia prevention and propose further system wide approaches to be assessed for effective dementia prevention and care delivery.

The US veteran population is aging and at increased risk of developing chronic neurodegenerative diseases such as Alzheimer's disease and related dementias. As of 2020, the Veterans Health Administration (VHA) provides comprehensive medical care for more than 9 million enrolled veterans at its 1,255 facilities, including 172 medical centers, and more than 1,074 outpatient clinics across the country. More than half of the patients in the VA are age 65 or older (1, 2). Changes in demographic characteristics such as more women and more diverse veteran populations may lead to a veteran population at higher risk of dementia. Several risks factors for dementia identified in the general population may be more prevalent in the Veteran population. Additionally, Veterans' risks for dementia can also be influenced by changes in VA policies on eligibility and enrollment criteria, and factors external to the VA. In this report, we summarize identification and prevalence of dementia in the VA, and current knowledge of demographic, health, and military related risk factors of dementia in the VA. The VA has experience in managing cardiovascular diseases, mental health, TBI, PTSD, and conditions that are common among veterans. We propose that VHA, the largest integrated health care system in the US, has promise in managing health risks that may impact dementia prevention and propose further system wide approaches to be assessed for effective dementia prevention and care delivery.

## Dementia Prevalence and Identification in the VA

Currently the VHA estimated dementia prevalence among VA patients age 65 and older at 9.6%. A recent meta-analyses of dementia prevalence studies reported a similar pooled prevalence rate of 10.1% in the VA (3). These estimates are broadly consistent with rates reported in the general population of 10.5% (4, 5). Prevalence of dementia among veterans is expected to increase dramatically in the coming decades (5–8). VHA Office of Policy & Planning projects a 22% increase in the number of VA Patients with Dementia (276,000–335,000) between 2020 and 2033 (5).

Several studies have estimated dementia prevalence among VA patients enrolled in specific programs or settings such as VA nursing homes and specialty mental health programs (9–11). Results from these studies are not directly comparable to those in the general veteran and non-veteran populations because of selection of patients into these programs and differences in patient characteristics (e.g., VA continues to care for a younger, more functionally disabled nursing home population than the non-VA population).

### Identification of Dementia in VA Administrative Data

Identification of dementia is complicated by many factors. There is substantial under-recognition, under-diagnosis, and under-recording of dementia in many systems. As much as 50% of dementia cases are un-identified in primary care settings (12, 13). One study in the VA suggested that many as 70% of the estimated veterans with dementia were not readily identified through their medical records (14). The VA has a sanctioned list of ICD codes intended to be used as the standard among all programs and services throughout the system for the purposes of dementia-related data analysis and reporting, including workload, cost, incidence, prevalence, forecasting (15), however it has not been used consistently across studies to identify dementia in the VA, making comparisons between studies difficult (9, 10).

In addition to VA care, older veterans have access to care outside the VA through almost-universal eligibility for Medicare, further complicating identification of dementia in the VA. Data from a national sample of VA patients with a formal diagnosis of dementia showed that approximately half of veteran with dementia used both VA and Medicare (16). There is substantial between systems coding disparities of dementia (17–19). In the general Medicare population, fewer than half of beneficiaries who have a clinical diagnosis of AD or related dementias have a recorded diagnosis in their claims (20). A study of VA patients age 65 and older who were enrolled in Medicare FFS in FY2013 showed that only 4.8% of veterans had a dementia diagnosis in VA data (4). Combining VA data with Medicare claims identified 7.4% of veterans with a dementia diagnosis, closer to the dementia prevalence rate of 9.6% among veterans estimated by VHA (21).

Historically, VA has served as a safety net to disabled and low income veterans and continues to serve a veteran population with much greater prevalence of chronic illness (4, 22–25). There are marked differences among veterans who seek care in both VA and Medicare compared to only in the VA. For example, reliance on VA health care was greatest among black veterans and those in high VA priority groups. Veterans with poorer health were most likely to rely on both VA and Medicare (26). Because of their cognitive impairment and dementia, these veterans may be particularly vulnerable to suffer from inferior care and under identification due to uncoordinated care across systems (27, 28). There is some evidence that dementia identification has been improving in recent years (29). In the VA, more consistent use of the VA sanctioned diagnoses along with those from Centers for Medicare and Medicaid Services (CMS) Chronic Condition Warehouse (CCW) (30) will be beneficial to improve dementia identification that is necessary to better care for dementia patients.

## Risks Factors for Dementia in the VA

### Demographic Factors

The veterans served by the VHA may be at higher risk for dementia than the general population for many reasons, including demographic, health, lifestyle, and military related factors. For example, the veteran population is becoming more diverse than the overall US population with higher percentages of African American and Latinos among veterans. These groups have historically had higher risk of dementia. According to a 2016 report, the prevalence of dementia is 26.6/1,000 among African Americans compared to 19.3/1,000 among non-Hispanic whites (31). Similar disparities between African Americans (19.3/1,000 years) and non-Hispanic whites (10.8/1,000) exist in the veteran population. The proportion of African Americans among younger veterans (those younger than 65) is nearly double that of those 65 or older and they represent an even higher proportion of patients in the VA, suggesting that this demographic risk will increase over the next few decades.

Most studies indicate increased risk of dementia in older women than men of the same age partly because older women typically had less education than older men (32). Although the proportion of women receiving health care in the VA is still low currently it is estimated to be increasing, suggesting potential for future increased risk of dementia in the VA. Several studies described below additionally indicate significant risk for dementia among women who are exposed to military related risks (33). One study reported significantly higher risk of developing dementia in 4 years in women veterans with TBI, PTSD, and depression, and those with more than one risk factor had a >2-fold risk of developing dementia (33).

It is well known that low education increases overall risk for dementia (34). This risk, recently described as an early life modifiable risk factor (32), may possibly be modifiable even later in life, although the evidence for that is not yet well-established. While veterans may have educational opportunities later in life that are not available in the general public, male veterans continue to lag behind non-veterans in obtaining a Bachelor's degree (35). While the opposite is true in female veterans they currently make up a small portion of the veteran population.

### Health Risks

Several comorbid conditions which have been associated with cognitive impairment and dementia appear to be more prevalent among veteran populations. There may be influences specific to veteran status that increase the risk of many conditions that contribute to cognitive impairment and dementia. For example, in a report from the Health and Retirement Study which examined individuals over 50 without heart disease at initial observation, self-reported veteran status was associated with increased risk of new onset heart disease compared non–veterans. The cohort, which is demographically representative and was tracked during the 20 year period from 1992 to 2012, identified veteran status as a significant risk factor even after controlling for demographic characteristics (age, gender, education, marital status), clinical characteristics (depression, hypertension, diabetes), and lifestyle (smoking, drinking, exercise). While this report did not examine the link between heart disease and cognitive impairment and dementia, others have reported that cardiovascular risks, such as hypertension, dyslipidemia, diabetes, metabolic syndrome, and obesity, all prevalent in veteran populations, are specifically associated with cognitive impairment and dementia (36, 37). The combined cardiovascular risk factors highlight the importance of clustering these factors which have identified dementia risk less readily observed when considered alone (38).

### Mental Health Risks

Mental health disorders such as depression, anxiety, and more recently, alcohol abuse syndromes have been identified as risks for dementia in the general population (32). All of these disorders are common among veterans. Specifically, a quarter of all Veterans seen in primary care at the Patient Aligned Care Teams (PACT) have 1 or more mental illness diagnosis, including depression (13.5%), substance use disorder (8.3%), anxiety disorder (4.8%), or other serious mental illness (3.7%) (39). Veterans also have relatively high estimates of dual diagnosis of these conditions with dementia. While in general the number of active psychiatric conditions decreases with age, they may actually be risks for cognitive impairment and dementia in later life. Depression and anxiety are well-recognized for increasing the risk of dementia and links *via* stress mechanisms have been proposed (40). These mental health conditions are also associated with increased vascular risks as are some of the most common medications used to treat these conditions. For example, studies have shown that depression, psychosis, and bipolar disorder were associated with increased risk of cardiovascular diseases (41). While some classes of medications exacerbated this risk, the effects of the conditions remained after treatment factors were controlled. The prevalence of cardiovascular diseases, metabolic risk factors, and mental health conditions creates a complex of circumstances that increase risks for dementia and make identifying dementia risks challenging.

### Military Related Risks: Head Trauma

Military experience can be associated with significant events that increase health risks in general and cognitive health risks in particular. Head injury, well-established as a risk factor for dementia among the general population (32), is common in military experience (42). Estimates in the recent past showed that over 1.5 million Iraq and Afghanistan veterans have suffered a traumatic brain injury (TBI) during deployment. Mild TBI (mTBI) such as that experienced with blast injury and possibly experienced as a mild concussion is likely to be under-reported (43, 44). A large national study of patients with TBI in the VA found that that mTBI even without loss of consciousness was associated with more than a 2-fold increase in the risk of dementia (45). There is growing evidence that mTBI is potentially causally association with a range of brain related outcomes including PTSD, depression, and other conditions. Preclinical work with animal models supports mechanisms by which mTBI can induce Post-Traumatic Stress Disorder (PTSD), and as discussed below, this may be a path to increased risk of dementia. Clinical work has yet to confirm these models.

### Military Related Risks: Post-traumatic Stress Disorder

The role of early and midlife stress related disorders as a risk factor for dementia have been studied both in military and non-military settings (46–50). Examining a national patient registry in Sweden, the presence of a stress related disorder, including PTSD, acute stress reaction, adjustment disorder, and other stress reactions was associated with a 31% increased risk in primary neurodegenerative diseases such as AD and an 80% increased risk in vascular neurodegenerative disease (51). A recent meta-analysis quantifying the association of PTSD and risk of dementia showed that PTSD as a strong risk factor for all-cause dementia in both military and non-military populations (52). In fact the study noted greater increase in dementia risk in the general population. However, the study also acknowledged greater increased risk for dementia in non-US Studies than studies from the US and also in studies with more than 50% females, making it difficult to interpret the difference between the general population and the military in US Veterans.

It is important to note that the prevalence of PTSD is likely greater in the military. Because of improved military health and battlefield survival from acute combat related injuries, veterans are at increased risk of PTSD and other mental health conditions which have been shown to be associated with developing chronic neurodegenerative diseases including AD and other dementias (7, 53). For example, both the National Comorbidity Survey (NCS) and its replication (NCS-R) estimated the lifetime prevalence of PTSD among adult Americans to be 6.8% (3.6%, among men and 9.7% among women) (54). The estimated lifetime prevalence of PTSD among Veterans on the other hand was 30.9% for men and 26.9% for women. 9.3% of Veterans seen in PACT have a diagnosis of PTSD (39). Among recent cohorts of veterans such as those returning from Iraq and Afghanistan, it is estimated that 17% experienced PTSD. Vietnam veterans have been found to have a 20–30% lifetime prevalence of combat-related PTSD. Since PTSD is a chronic condition, appearing in some cases many years after return, it is not surprising that even among World War II veterans persistence of PTSD after 45 years is estimated at least at 12%. One study reported that veterans with an ICD diagnosis of PTSD had cumulative incidence rate of 10.6% for dementia, while those without PTSD had a rate of 6.6% (46). Neither demographic nor medical comorbidities have been shown to modify the impact of PTSD on dementia risk. This result has been replicated in smaller studies examining older veterans (47), and the effect does not appear to be modified by the use of psychotropic medications (49). Even if the increased risk for dementia is lower in veterans than in the general public these differences in prevalence of PTSD may lead to a greater number of cases.

## Potential for Managing Risks to Cognitive Health

As an integrated health care system, the VHA has opportunities to provide management of health conditions that may reduce the risk of cognitive impairment and dementia. Quality management indicators are powerful tools to encourage facilities to systematically screen for important co-morbidities and to meet certain rates of service delivery. Availability of “wrap around” services can be used to overcome considerable barriers in disease management. Several examples of such programs in the VA include the Self-Management to Prevent (STOP) Stroke Program to help veterans at risk for stroke and stroke recurrence. As a response to patients' initial feedback indicating desire for more in-depth nutrition education in the program, a teleconference program was developed to include the Dietary Approaches to Stop Hypertension (DASH) diet nutrition concepts and transportation services to overcome compliance and retention barriers. Well-developed education and training programs are integrated in clinical practice guidelines and have been used to outreach with health management through community based Veteran Service Groups (VSO) such as American Legion and veteran of Foreign Wars. This is exemplified in POWER (Posts Working for Veterans' Health), an intervention of peer leaders from VSOs designed to improve and facilitate self-care behaviors that contribute to blood pressure control (55). A system wide engagement program in risk reduction is the *MOVE*! Weight-Management Program, which was piloted in 17 VAMCs successfully implemented nationally, with more than 100,000 patients having participated in more than 500,000 visits.

One way to consider risk management is through early detection and screening. While the US Preventive Services Task Force (USPSTF) currently finds inconclusive evidence to recommend routine asymptomatic cognitive screening in the general population (56), it is possible that the high risk for cognitive impairment and dementia in the VA may tilt risk-beneficial ratio in this population to make screening worthy of consideration. In an early study carried out in 7 VA medical centers assessing the feasibility of cognitive screening in older veterans presenting for routine primary care, veterans overwhelmingly accepted cognitive screening administered on the day of a routine primary care clinic visit, with almost 97% agreeing to screening (14). With its experience in comprehensive screening programs including TBI, a risk factor for dementia, the VA has potential in implementing system wide approaches in managing health risks that impact dementia prevention care delivery.

While these studies have focused on translating science to care delivery, none have yet to examine cognitive benefits that might have been gained. In fact, the lower risk of dementia from PTSD among veterans within the VA health care system compared to the general population may partly be the result of availability and of delivery of targeted treatments in the VA for this condition which may not be available to the general public. Transformational care delivery is possible and the opportunity to assess the impact in reduction of cognitive impairment and dementia is within reach.

## Conclusions

Risk of dementia are high among veterans and are influenced by many factors, including changes in veterans' demographic and socioeconomic characteristics, clinical, health, lifestyle features, and military related risk factors. Compared to those without dementia, veterans with dementia are older, sicker, and more likely to rely on care within and outside the VA. Veterans with dementia who also seek care outside the VA may be particularly vulnerable as multiple sources of care can lead to fragmentation of care that may negatively impact the quality of care veterans receive and their health outcomes.

The comprehensive nature of health care delivery within an integrated system such as the VHA provides opportunities to improve both quality of care and cost effectiveness of care. System wide approaches to screening, behavioral management, and disease prevention have potential to mitigate risks of dementia. Interventions to reduce fragmentation of services and integrate care across settings can improve both quality of care and cost effectiveness. These approaches have had successes with other chronic conditions and research in cognitive health and dementia care delivery may provide similar best practices for aging veterans.

## Author Contributions

MS and CZ substantial contributions to conception or design of the work, and acquisition, analysis, interpretation of data for the work, drafting of the work, revising it critically for important intellectual content, final approval of the version to be published, and agreement to be accountable for all aspects of the work in ensuring that questions related to the accuracy or integrity of any part of the work are appropriately investigated and resolved.

## Conflict of Interest

The authors declare that the research was conducted in the absence of any commercial or financial relationships that could be construed as a potential conflict of interest.
